# Assessment of Formalin-Fixed Bovine Larynx Asymmetry Using Geometric Morphometry

**DOI:** 10.1155/tswj/8386223

**Published:** 2025-02-04

**Authors:** Arcesio Salamanca-Carreño, Pere M. Parés-Casanova, Mauricio Vélez-Terranova, Néstor Ismael Monroy-Ochoa

**Affiliations:** ^1^Faculty of Veterinary Medicine and Zootechnics, Cooperative University of Colombia, Villavicencio 50001, Colombia; ^2^Bromatology Department, Oberta University of Catalonia, Barcelona 08018, Spain; ^3^Faculty of Agricultural Sciences, National University of Colombia, Palmira 763531, Colombia

**Keywords:** directional asymmetry, larynx size, morphology, morphometric profile, shape asymmetry

## Abstract

The vibrations of the larynx produce the voice. Little is known about morphology and the size of cattle larynx. The aim of the study was to quantify the shape asymmetry of the larynx in calves obtained *postmortem* using a geometric morphometric approach. A sample of 14 larynges from calves (in an age range 335–625 days) belonging to “Bruna dels Pirineus” breed and its F1 crosses was obtained in an abattoir during the first semester of 2021. Laringes were dissected and preserved in the laboratory until their photograph was taken Landmark sets were superimposed on images of fixed transverse sections of larynges. Using geometric morphometric techniques, we analyzed the presence of asymmetries. Asymmetry between sexes was assessed by means of a Canonical Variate Analysis on regression residuals of asymmetric components using the Mahalanobis distance, which assumes a lack of isotropy. Significant differences in the morphometric profile were found between the right and left larynx and between the sexes (*p* < 0.05). Results demonstrate differences between right and left larynx sides as directional asymmetry, at least in fixed structures. Our findings provide a foundation for quantifying the possible contribution of larynx asymmetry among cattle. This study can be considered the first to detect larynx asymmetries in cattle. Studies with a larger sample size are needed to validate the information in large populations. One limitation is that the present study does not have samples from adult animals, so it would be interesting to know if the results are similar among them.

## 1. Introduction

The larynx is a part of the respiratory system [1] and protects the respiratory tract during swallowing, respiration, and voice production, and provides a mechanism for organisms to seal the airways, and hold their breath [1, 2]. The larynx is important for communication between members of the same species and evolutionary studies, as in the case of anuran species [2, 3]. Voice production occurs as a result of vibrations of the larynx [1, 4]. Although there are descriptions on the form and constitution of the cartilaginous muscle of the larynx of animals, there are few studies on signs of sexual dimorphism in the larynx in domestic or wild mammals [5]. Larynx vibration facilitates the production of sounds that exhibit reduced environmental attenuation [6]. “The size and composition of larynx” appears to contribute to species specific acoustic properties [7, 8]. Little is known about size and morphology of cattle larynx. Vocal organ morphology represents a “complex shape and the absence of homologous landmarks pose major challenges in its geometrical analysis” [9]. In older females, vocal cord contact increases, while in males it decreases with age [10, 11].

The association between larynx length, elasticity, and body size has been described in several mammal species, such as human, red deer, horses, and Rocky Mountain elk [4, 12]. Moreover, in humans the larynx is sexually dimorphic [13, 14] as well as in rats [14]. Despite this observed sexual dimorphism, little is known about the sexual dimorphism of the thyroarytenoid, the main muscle of the larynx [14]. The literature suggests that the number of muscle fibers decreases in the thyroarytenoid, and differences have been found between the thyroarytenoid muscles of male and female rats in old age [15]. But sex differences in larynx contact area during sound production have not been studied for any animal, at least in authors' knowledge. We therefore hypothesized that there would be noticeable shape changes in the larynx associated with functional vocalizations differences among genders in cattle.

Although geometric morphometrics (GM) is not a novel technique for the study of form in structures, it has been scarcely applied to the study of soft tissues [16]. Nor has it been applied to the study of soft tissue sexual dimorphism [17]. So, its application to the analysis of shape variation for larynx is totally novel. In this research, we apply GM techniques to the study of larynx in a sample of meat calves, with the purpose to study form (size + shape) of both sides of larynx, and test the following research questions:

1. Is there any asymmetry (directional or fluctuating) in the bovine larynx?
2. Are there asymmetric differences (directional) between the sexes?

The aim of the present study was to assess anatomical asymmetry using GM techniques of the formalin-fixed larynx in “Bruna dels Pirineus” breed. A preprints.org has previously been published [18].

## 2. Materials and Methods

### 2.1. Sample

**Figure 1:**
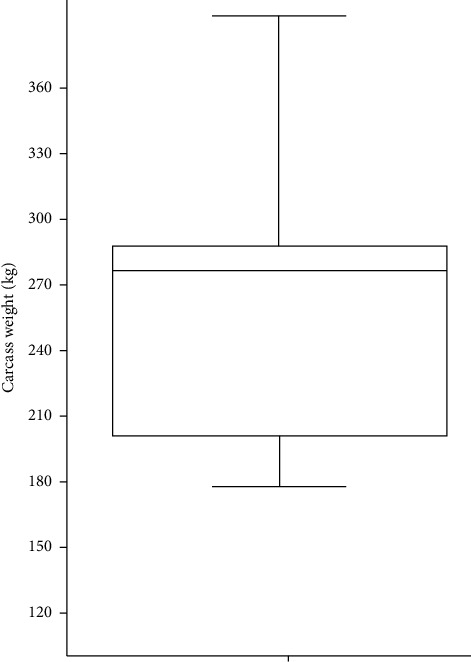
Box plot for hot carcass weight 263.3 ± 53.26 kg.

A sample of 14 larynges from calves belonging to “Bruna dels Pirineus” breed and its F1 crosses (cross with another meat breed) was obtained in an abattoir during the first semester of 2021. “Bruna dels Pirineus” is a breed from Catalonia (NE Spain) with meat purposes and managed under semiextensive conditions mainly on the Pyrenean and Prepyrenean area. Sample included 6 females and 8 males in an age range 335–625 days and a mean of 263.3 ± 53.26 kg for hot carcass weight (Figure 1), which is the commercial age demanded by the Catalan market. The age was calculated according to official individual documents (mandatory). No animal presented anomalies on *antemortem* inspection. Larynx samples were obtained from fresh carcasses. The samples included all the pieces and were never fragmented.

**Figure 2:**
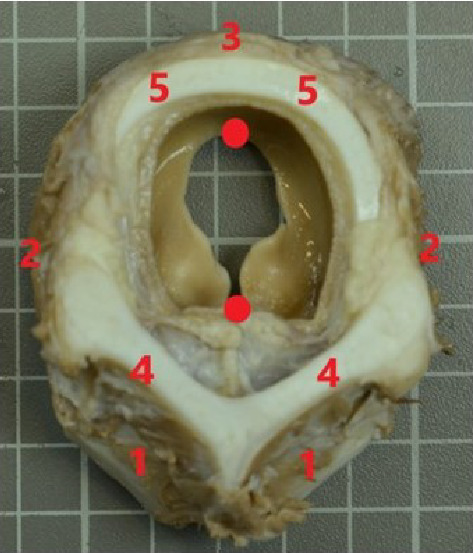
Transversal section (rostral view) at the level of larynx. A scale was included and placed parallel to the face plane. Red points mark the mid-sagittal landmarks. (1) Cricothyroid muscle; (2) arytenoid muscle; (3) cricoaritenoid muscle; (4) lamina of thyroid cartilage; 5: lamina of cricoid cartilage.

At the laboratory, fresh larynges were dissected and were preserved in buffered formaldehyde at least for a minimum of 72 h 4% until they were photographed. A transversal cut at the level of larynx was obtained and fixed in a standard plane and pictures were then taken using a digital camera (Nikon D1500) equipped with a lens (Nikon DX de 18–105 mm). A scale was included and placed parallel to the face plane (Figure 2).

### 2.2. Comparison Between Sexes

Age and hot carcass weight between sexes were compared using the Mann–Whitney *U* test.

### 2.3. GM

A set 2 midsagittal landmarks and 30 semilandmarks per side were selected. Location and digitalization were performed with TpsDig v. 2.16 [19]. Digitalization was done in two separate sessions. Semilandmarks are points on curves for which their exact position cannot be determined using anatomical criteria. They were ulteriorly transformed to landmarks with the program TpsUtility v. 1.70 [19]. Statistical analyses were done using standard procedures [20].

All coordinates were then aligned using a Procrustes superimposition, thus eliminating non-shape information. Size (expressed as Centroid Size, CS) was estimated as “the square root of the sum of squared distances from the centroid of a landmark configuration” [20]. To perform size correction, the allometric influence of size on shape was estimated by regressing the shape scores on the CS log transformed.

It has been noted “that measurement error is a confounding factor in the assessment of fluctuating asymmetry” [21–23]. Therefore, all pictures were digitized twice to estimate inter-observer error. A shape Procrustes ANOVA to analyze the variation in total shape and to examine the mean squares proportion of measurement error to the overall variation was performed. The symmetric component is the variation among individuals in the consensus. “The asymmetric component is the squared distances between the original configurations from this symmetric component” [22, 23]. Since Procrustes ANOVA assumes an isotropic variation around the landmark configuration, a Multivariate Analysis of Variance (MANOVA) test with a Pillai's trace and the associated parametric *p* value to assess the statistical significance was performed. Finally, the asymmetry between sexes was assessed by means of a Canonical Variate Analysis (CVA) on regression residuals of asymmetric components using the Mahalanobis distance, which assumes a lack of isotropy.

Symmetry was considered as object symmetry for example, as a single structure which is identical according to a mid-sagittal plane [22]. To analyze the total shape variation of the entire sample into components of symmetric variation (“Individuals”), directional asymmetry (“Side”), and fluctuating asymmetry (“Individual by Side interaction”), a Procrustes ANOVA was calculated.

Analyses were carried out with MorphoJ v. 1.07a software [24]. A *p* value of < 0.05 was considered statistically significant.

## 3. Results

### 3.1. Comparison Between Sexes

Carcass weight was statistically higher (*U* = 42; *p*=0.00123) among males (220.2 ± 46.7 kg) than among females (177.8 ± 42.4 kg), but age did not differ between sexes (*U* = 116; *p*=0.664).

### 3.2. Allometry

**Figure 3:**
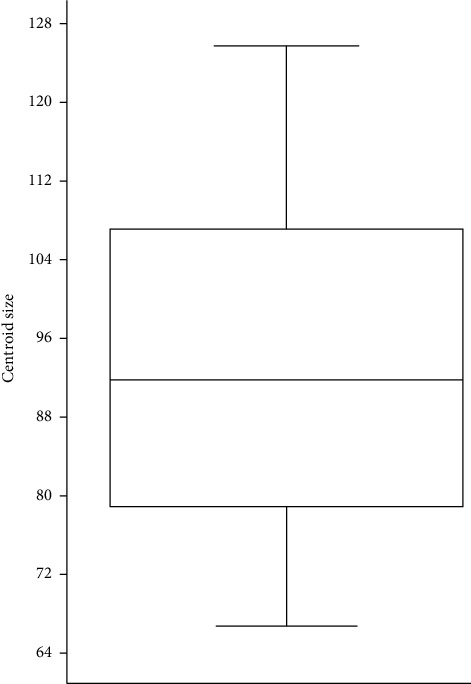
Box plot for centroid sizes.

The contribution of global size variation was 17.52% (*p*=0.0008; 10,000 randomization rounds). A clear correlation between centroid size (Figure 3) and carcass weight was found (*r*^2^ = 0.527; *p*=0.0019, data log-transformed).

### 3.3. ANOVA

Measurement error was 4.5% (Table 1). ANOVA also showed a significant effect for DA (47.6%) and FA (9.5%, mainly on the former (Table 1).

### 3.4. Shape Changes

**Figure 4:**
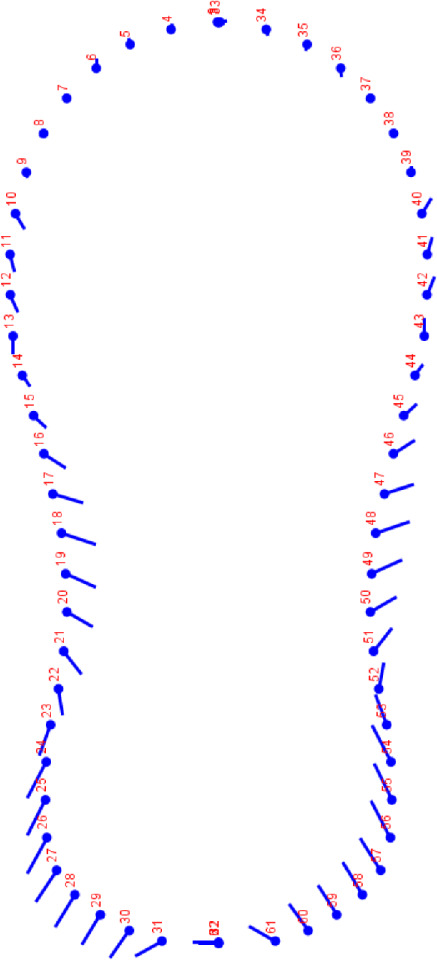
Deformation grid depicted a clear right-biased basal and left-biased central cartilages based on the direction of vectors. Rostral view.

Deformation grids helped visualize the shifts in aligned coordinates from one shape to another and it depicted a clear directional bias (toward left on the lateral parts and toward right on the ventral parts) (Figure 4). Significant differences in the morphometric profile were found between the right and left larynx, and between the sexes (*p* < 0.05).

### 3.5. CVA

The asymmetric components were significantly different among sexes (*p* < 0.0001; 10,000 permutation rounds).

## 4. Discussion and Conclusion

In this study, significant differences were found between the sexes and in the morphometric profile between the left and right part of the larynx. Laryngeal sexual dimorphism is recognized in humans [25] and reindeer [26] but has not been found in dogs [27], in horses [28] or pigs [29]. In the larynx of adult rats, researchers have also found sexual dimorphism in function and structure, with lower frequency of vocalization in males [14].

At the onset of normal larynx vibration, the larynx are adducted [30] (their displacement is away from the midline in symmetric movements), so our results signal an asymmetric vibration, with a possible lack of equivalence with the shape properties of the larynx. In fact, it has been presumed “that laryngeal asymmetry (either in mass or tension) causes irregular vibrations” [31]. The laryngeal tissue, in particular the cartilages is sensitive to testosterone leading to a larger overall growth in males, and this in turn leads to larynx size sexual dimorphism in some species [4], and this would explain the detected sexual dimorphism, as males were larger than females. Asymmetry was correlated with larynx size, which in turn appeared to be correlated with body weight (e.g., more asymmetry with body weight). Other studies report that the higher the body height, “the laryngeal measurements were statistically significant,” and higher for males [32]. Variations in laryngeal structure may explain much of the interpopulation signal differences [33], body size and phylogeny [34] and sexual differences. Efforts to quantify laryngeal morphology have been hampered by its complex structure [9], which poses challenges for comparative analyses [34]. However, imaging technology has made morphological reconstruction possible in such structures [4].

The main limitation of the present study is that the sample size was limited to 14 larynges. As formaldehyde fixation can induce shrinkage, the question remains how different a fixed larynx is from a fresh one. Our results do not necessarily correspond to the situation of live cattle, but we have just intended an approximation of comparison between sides, so detected asymmetry could be found in fresh structures as well.

In this study, calves were used because they are the ones that usually arrive at the slaughterhouse. Evidently, sampling in other factories could have enlarged the sample and the age range, but it was not possible due to sanitary restrictions. Likewise, there is an important lack of bovine larynx anatomy. This reinforces the importance and the novelty of our study.

This study can be considered the first to detect larynx asymmetries in bovine livestock. Further study involving a larger sample size is needed to validate this information in a larger population. Regarding other limitations, the present study has not sampled adult animals, so it would be worth investigating if results are similar among full-grown animals.

## Figures and Tables

**Table 1:** Measurement error procrustes ANOVA for size and shape of larynx for calves (*n* = 14), with a significant effect DA (directional asymmetry) and FA (fluctuating asymmetry) for shape.

Effects	SS	MS	Df	*F*	*p*
*Size*					
Individuals	10,064.73	6.290.458	16	179.02	< 0.0001
Error	5.622.012	3.513.757	16	0.04	1
Residual	3.486.127	8.715.316	4		

*Shape*					
Individuals	0.899,359	0.000,937	960	4.14	< 0.0001
DA	0.068,079	0.001,135	60	5.01	< 0.0001
FA	0.217,214	0.000,226	960	2.09	< 0.0001
Error	0.207,778	0.000,108	1920	−4.48	NaN
Residual	−0.011,600	−2.4 × 10^−05^	480		

*Note:* Sums squares (SS) and mean squares (MS) are in units of procrustes distances (dimensionless).

## Data Availability

Data are available upon reasonable request to the second author.
